# Clinical results of mean GTV dose optimized robotic guided SBRT for liver metastases

**DOI:** 10.1186/s13014-016-0652-4

**Published:** 2016-05-28

**Authors:** Nicolaus Andratschke, Alan Parys, Susanne Stadtfeld, Stefan Wurster, Stefan Huttenlocher, Detlef Imhoff, Müjdat Yildirim, Dirk Rades, Claus Michael Rödel, Jürgen Dunst, Guido Hildebrandt, Oliver Blanck

**Affiliations:** Department of Radiation Oncology, University Medicine Rostock, Rostock, Germany; Department of Radiation Oncology, University Hospital Zürich, Zürich, Switzerland; Department of Radiation Oncology, University Hospital Frankfurt, Frankfurt, Germany; Department of Radiation Oncology, University Medicine Greifswald, Greifswald, Germany; Saphir Radiosurgery Center, Frankfurt, Güstrow Germany; Department of Radiation Oncology, University Medical Center Schleswig-Holstein, Lübeck, Germany; Department of Radiation Oncology, University Medical Center Schleswig-Holstein, Kiel, Germany; Department of Radiation Oncology, University Hospital Copenhagen, Copenhagen, Denmark

**Keywords:** Liver metastases, Gross tumor volume optimization, Stereotactic body radiation therapy, CyberKnife robotic radiosurgery

## Abstract

**Background:**

We retrospectively evaluated the efficacy and toxicity of gross tumor volume (GTV) mean-dose-optimized and real-time motion-compensated robotic stereotactic body radiation therapy (SBRT) in the treatment of liver metastases.

**Methods:**

Between March 2011 and July 2015, 52 patients were treated with SBRT for a total of 91 liver metastases (one to four metastases per patient) with a median GTV volume of 12 cc (min 1 cc, max 372 cc). The optimization of mean GTV dose was prioritized during treatment planning at the potential cost of planning target volume (PTV) coverage reduction while adhering to safe normal tissue constraints. The delivered median GTV biological effective dose (BED_10_) was 142.1 Gy_10_ (range, 60.2 Gy_10_ –165.3 Gy_10_) and the prescribed PTV BED_10_ ranged from 40.6 Gy_10_ to 112.5 Gy_10_ (median, 86.1 Gy_10_). We analyzed local control (LC), progression-free interval (PFI), overall survival (OS), and toxicity.

**Results:**

Median follow-up was 17 months (range, 2–49 months). The 2-year actuarial LC, PFI, and OS rates were 82.1, 17.7, and 45.0 %, and the median PFI and OS were 9 and 23 months, respectively. In univariate analysis histology (*p* < 0.001), PTV prescription BED_10_ (HR 0.95, CI 0.91–0.98, *p* = 0.002) and GTV mean BED_10_ (HR 0.975, CI 0.954–0.996, *p* = 0.011) were predictive for LC. Multivariate analysis showed that only extrahepatic disease status at time of treatment was a significant factor (*p* = 0.033 and *p* = 0.009, respectively) for PFI and OS. Acute nausea or fatigue grade 1 was observed in 24.1 % of the patients and only 1 patient (1.9 %) had a side effect of grade ≥ 2.

**Conclusions:**

Robotic real-time motion-compensated SBRT is a safe and effective treatment for one to four liver metastases. Reducing the PTV prescription dose and keeping a high mean GTV dose allowed the reduction of toxicity while maintaining a high local control probability for the treated lesions.

## Background

Liver metastases are common for disseminated cancer disease, especially for primary tumor sites in the gastrointestinal tract [1, 2]. The liver is even amongst the first and possibly only site of failure in patients with colorectal cancer. Synchronous liver metastases are found in 15–25 % of these patients at the time of primary diagnosis, and another 20 % of patients will develop metachronous liver metastases. Hepatic resection represents the gold standard for local therapy [3, 4], although only 10–20 % of all patients are candidates for surgical resection, depending on tumor size, localization, or liver function [4, 5]. Patients with untreated hepatic metastases have a very poor prognosis with an actuarial 5-year overall survival (OS) of < 3 % and a mean OS of 4–12 months [6].

In comparison the 5-year actuarial OS for patients with resected liver metastases ranges between 32 and 74 % [7–9]. Because of the large amount of inoperable patients and because hepatic recurrences occur in nearly two thirds of the patients after surgical resection [10], several alternative or complementary therapies have been established such as radiofrequency ablation (RFA) [11], trans-arterial chemoembolization (TACE) [12], laser-induced thermotherapy (LITT) [13], selective internal radiation therapy (SIRT) [14] or stereotactic body radiation therapy (SBRT) [15–21].

New developments in SBRT technology, such as active-breathing-controlled tumor localization [21] or real-time tumor-tracking technology (CyberKnife®, Accuray Incorporated, Sunnyvale, CA, USA) [22, 23], have allowed the application of high radiation doses within the gross tumor volume (GTV) while applying minimal safety margins aiming at maximal sparing of surrounding normal tissue. This is especially challenging in presence of large tumor motion which is predominantly found in the lower part of the lung and in the liver. Besides, a dose response relationship for local control of liver metastases has been reported and effective SBRT of liver metastases should aim at delivering high biologically effective radiation doses (BED) within the tumor [15, 17, 24].

Nevertheless, using the small CyberKnife beams, this task is not always straightforward as compared to standard isocentric SBRT with gantry-based linear accelerators. Therefore, our approach was to optimize robotic radiosurgery treatment planning to maximize the dose within the GTV at the potential cost of planning target volume (PTV) coverage while adhering to safe normal tissue constraints. The aim of our retrospective analysis for local control (LC), progression-free interval (PFI), overall survival (OS), and toxicity was to validate our GTV-optimized treatment approach in comparison to other published studies.

## Methods

### Patient characteristics

This retrospective analysis was approved by the respective ethics committees of the treating centers. Between March 2011 and July 2015, 52 patients with a total of 91 metastatic liver lesions (one to four metastases per patient) were treated with SBRT using the CyberKnife real-time tracking system at two centers performing radiosurgery (Table 1). Three patients had two and three patients had three repeat SBRT procedures for new liver metastases which developed during follow-up, and were included in the analysis. All patients’ primary tumors were controlled at the time of SBRT treatment and SBRT was chosen as first therapeutic option at initial diagnosis of liver metastases in 26.9 %, at the diagnosis of recurrent metastases after local therapy in 9.6 %, or at the diagnosis of new metastases after systemic failure in 63.5 % of the patients. Furthermore, 63.5 % of the patients had no extrahepatic disease, while 22.8 % had stable extrahepatic disease, and 13.7 % underwent simultaneous treatment of extrahepatic disease at the time of SBRT, mostly consisting of lung, bone or lymph node metastases. All patients were considered oligometastatic having less than five metastatic sites. Median baseline Karnofsky Index was 90 % (range, 60–100 %) and median GTV volume was 12 cc (min 1 cc and max 372 cc).

**Table 1:** Patient, tumor and treatment characteristics

		Total	%
Patients		52	
Lesions		91	
Gender			
	Male	30	57.7
	Female	22	42.3
Age	Median (range) in years	62	(37–89)
Karnofsky-Index	Median (range) in %	90	(60–100)
Primary Tumor			
	Colorectal	22	42.3
	Breast	4	7.7
	Lung	4	7.7
	Other	22	42.3
Extra-hepatic Disease			
	No	33	63.5
	Yes	19	36.5
Time of SBRT			
	At first diagnosis of metastases	14	26.9
	After local therapy	5	9.6
	After chemotherapy	33	63.5
Time to SBRT	Median (range) in months	7	(0–56)
Lesions per Treatment			
	1	39	63.9
	2	17	27.9
	3	2	3.3
	4	3	4.9
	Total		
Repeat Procedures			
	2	3	5.8
	3	3	5.8
GTV Volume	Median (range) in cc	12	(1–372)
Max GTV Dimension	Median (range) in cm	2.8	(1.2–8.9)
PTV Dose per Lesion			
	3 × 15 Gy	21	23.0
	3 × 14 Gy	10	11.0
	3 × 13 Gy	15	20.9
	3 × 12 Gy	14	15.4
	3 × 8-10 Gy	10	11.0
	4 × 7-10 Gy	11	12.1
	5 × 6-9 Gy	10	11.0
PTV Prescription BED	Median (range) in Gy	86.1	(40.6–112.5)
GTV Mean BED	Median (range) in Gy	142.1	(60.2–165.3)

*GTV* Gross Tumor Volume, *PTV* Planning Target Volume, *BED* Biological Effective Dose

### Fiducial implantation

Prior to treatment either GoldAnchor™ (Naslund Medical AB, Huddinge, Sweden) or solid gold fiducial markers (IZI Medical Products, Owings Mills, MD, USA) were implanted as close to the lesion as possible with computer tomography (CT) or ultrasound (US) guidance. Depending on the size, shape and number of the lesions, one (e.g., for a small spherical lesion) to five (e.g., for multiple lesions) fiducials were implanted.

### Treatment planning

Treatment planning was performed on standard non-contrast-enhanced CT scans at regular end expiration breath hold with 1.5-mm slice thickness. The planning CT was fused with a T2- and multiple T1-weighted magnetic resonance images (MRI) at 0–20 min after injection of intravenous contrast agent (Gadovist, Bayer, Germany). When MRI was not available (e.g., due to pacemaker) a secondary contrast-enhanced CT was additionally used for treatment planning. A composite GTV was defined as the sum of the GTVs contoured on each of the fused CT or MRI images according to a previously published CyberKnife protocol [24]. The clinical target volume (CTV) consisted of the GTV with an expansion of 5 mm in all directions within the liver (excluding extension beyond liver parenchyma) to encompass microscopic tumor spread [25]. The PTV included the CTV and an expansion of 3 mm in all directions to encompass the targeting uncertainties for the CyberKnife system [26, 27].

**Fig. 1:**
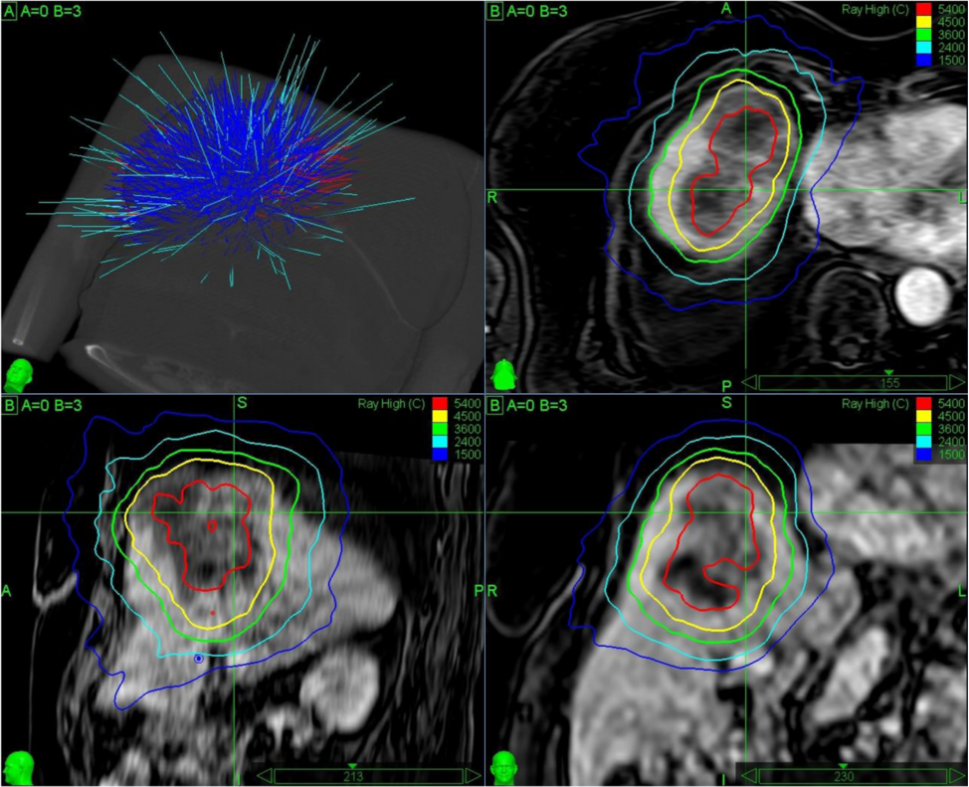
CyberKnife treatment plan of a 226 cc metastasis in the upper liver overlaid on contrast enhanced T1-weighted MRI in axial (*upper right*), sagittal (*lower left*), and coronal (lower right) view. The prescription dose to the planning target volume was lowered to 3 × 12 Gy (BED_10_ = 79.2 Gy_10_) to the 60 % isodose due to the size and critical organ constraints (i.e., the heart). The mean gross tumor volume dose was optimized to 140 Gy_10_. Twenty-eight months after treatment, the patient was alive and had complete response with no further development of metastases in the meantime. Upper left: Planning CT with final CyberKnife treatment beams (cyan) from the initially generated beam assortment (*blue*/*red*)

Beam optimization was performed using the MultiPlan® (Accuray) treatment planning software (version 3.5 and 4.5) and the Sequential Multi-Objective Optimizer [28] according to the consensus guidelines for treatment planning for robotic radiosurgery [29]. The main objectives for optimization were to maximize the GTV mean dose above 3 × 18 Gy (BED_10_ = 151.2 Gy_10_), to cover 95 % of the PTV with 3 × 15 Gy with a maximum dose of 3 × 20 Gy, and to minimize all critical structures according to the ALARA (As Low As Reasonably Achievable) principle. If due to critical organ constraints [30] 3 × 15 Gy was not achievable for the PTV, the prescription dose was lowered (3 × 8–14 Gy) in 34.1 % of the cases and/or more protracted fractionation schedules were used (4–5 fractions) in 23.1 % of the cases. The aim in these cases was to maintain a high GTV mean dose above 3 × 18 Gy (BED_10_ = 151.2 Gy_10_) while not raising the maximum dose of 3 × 20 Gy (BED_10_ = 180 Gy_10_). In other words, whenever the PTV prescription dose was lowered, we compensated by generating steeper dose gradients in the CTV/PTV margin zone outside the GTV (Fig. 1). The final prescribed dose to the PTV ranged between 23 Gy and 45 Gy (median 39 Gy) to the 60–83 % isodose (median 75 %) and the mean GTV dose ranged between 33 Gy and 57 Gy (median 52.5 Gy) resulting in a median BED_10_ of 86.1 Gy_10_ (min 40.6 Gy_10_ and max 112.5 Gy_10_) surrounding 95 % of the PTV and of 142.1 Gy_10_ (min 60.2 Gy_10_ and max 165.3 Gy_10_) for the mean GTV.

### Treatment delivery

SBRT was delivered using the Synchrony® Respiratory Tracking System (Accuray) (versions 8.5 and 9.5). All patients were loosely immobilized using a custom made vacuum mattress (HEK Medical, Germany) and initially aligned using the spinal vertebra closest to the lesions. Respiratory motion was initially modeled, updated during treatment, and compensated during beam-on periods using the prediction of two to three LED chest markers which were correlated to one to five previously implanted gold fiducial markers detected on orthogonal x-ray imaging [22, 23, 31]. Combined average tracking errors (i.e., correlation and predictions errors) were kept below 1 mm and average rotation errors were minimized by aligning the patient to the average breathing phase, if rotation was detectable by the CyberKnife system. Median fraction treatment time was 41 min (range 18–76 min), excluding setup time.

### Follow-up and statistical analysis

All patients were observed 6 weeks after the end of their SBRT treatment and every 3 months thereafter. Every follow-up included the recording of possible adverse events according to the Common Terminology Criteria for Adverse Events (CTCAE, Version 4.03) for acute toxicity and the Radiation Therapy Oncology Group (RTOG) and European Organization for Research and Treatment of Cancer (EORTC) criteria for late toxicity. Imaging during follow-up was kept similar to the planning imaging (contrast-enhanced MRI or CT where applicable) and evaluated using the Response Evaluation Criteria In Solid Tumors (RECIST) with a dedicated focus on differentiation of radiation effects in the liver versus actual tumor growth.

In our statistical analysis, we evaluated local control (LC), progression-free interval (PFI), overall survival (OS) and toxicity. Local control was defined as complete remission (CR), partial remission (PR) or stable tumor size (ST) and was independently confirmed by PET imaging when CT or MRI gave suspicious but inconclusive results. All time points for LC, PFI and OS were calculated from the end of SBRT treatment to the respective event; death of any cause was the endpoint for OS. In case of multiple SBRT treatments, PFI and OS were calculated from the end of the first SBRT series and for LC each lesion was observed separately from the end of the respective SBRT treatment. In case of local recurrence, time to first description of suspected recurrence (back dating) was recorded. Surviving patients without a disease progression were censored at last follow-up. All curves were estimated using the Kaplan-Meier method.

The comparison of different patient or dosimetry groups was performed using the log-rank-test for the univariate and using cox-regression for the multivariate analysis. For LC histology, GTV volume, PTV prescription dose expressed as BED and GTV mean dose expressed as BED and for PFI and OS gender, age, GTV volume, cumulative GTV volume, Karnofsky performance status, histology, previous systemic treatment, extrahepatic tumor status and number of metastases were used as variables in the univariate analysis. For the indication of statistical significance a p-value of ≤ 0.05 was considered. Kaplan-Meier survival estimates and curves were calculated using the statistical program SPSS (Version 20.0, IBM, Armonk, USA). Cox regression survival analysis was performed using the R programming language for statistical computing (Version 3.2.3, The R Foundation for Statistical Computing, Vienna, Austria).

## Results

At the time of analysis, the median follow-up for all patients after each SBRT treatment was 17 months (range, 2–49 months). Censoring for 1 year was 37.4 % and for 2 years was 64.8 %.

### Local control

**Fig. 2:**
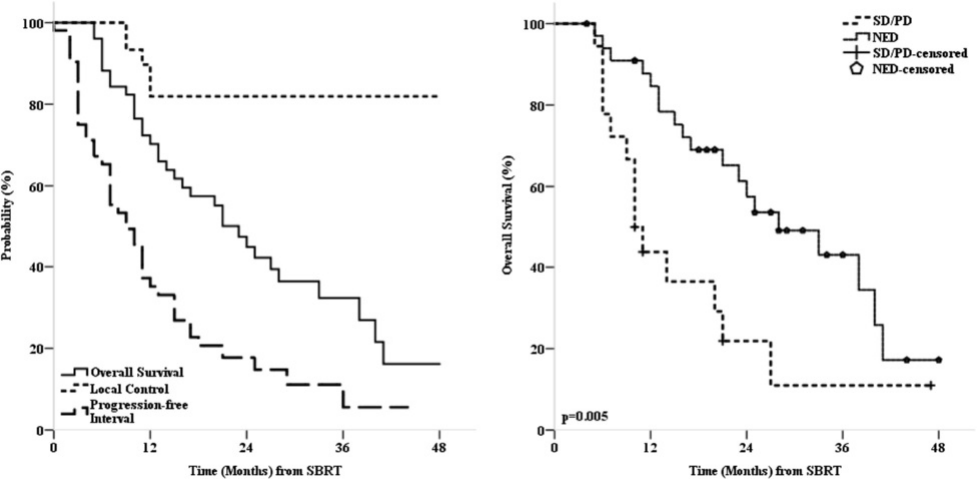
*Left*: Kaplan-Meier estimates for local control progression-free interval and overall survival. *Right*: Overall survival stratified by extrahepatic disease status, SD = stable disease, PD = progressive disease, NED = no evidence of disease

**Fig. 3:**
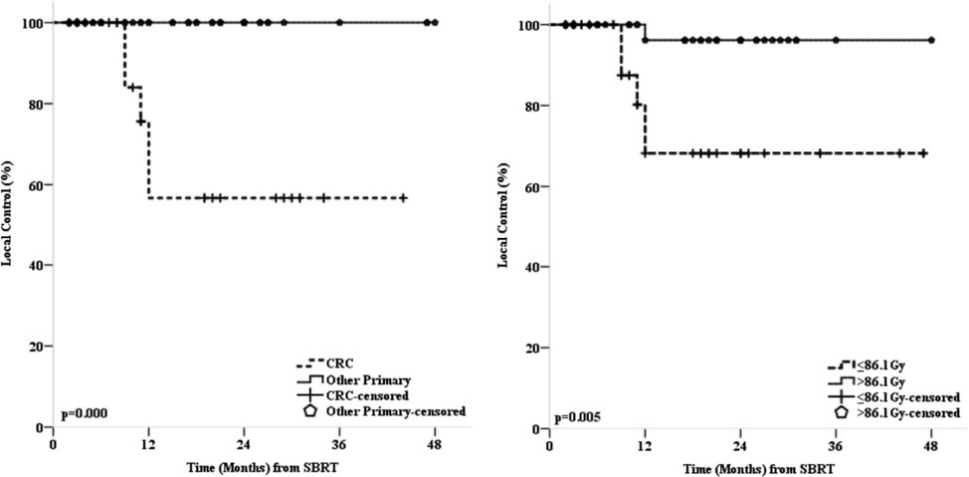
*Left*: Kaplan-Meier estimates for local control stratified by primary tumor site. *Right*: Kaplan-Meier estimates for local control stratified by planning target volume biologically effective prescription dose

Local control (LC) was analyzed per treated lesions (*n* = 91). The overall crude LC at time of analysis was 89.0 %. The 2-year actuarial LC rate for all treated liver metastases was 82.1 % (Fig. 2). All 10 local failures occurred in patients with primary colorectal cancer (Fig. 3). Nine out of the 10 local failures occurred in two patients (one patient with two treatments of one and four metastases and one patient with one treatment of four metastases). Two out of ten local failures were marginal recurrences after previous SBRT treatment (re-treatment recurrences), and the remaining eight were from two patients who were treated for four metastases each and experienced simultaneous in-field recurrences. Five recurrences were re-treated with SBRT (two patients with one and four treated metastases), one recurrence was resected and four recurrences (one patient with four treated metastases) were treated with further chemotherapy only. In univariate analysis histology (colorectal cancer vs. other primary cancers, *p* < 0.001), PTV prescription BED_10_ (Hazard Ratio HR 0.95, 95 % CI 0.91–0.98, *p* = 0.002) and GTV mean BED_10_ (HR 0.975, CI 0.954–0.996, *p* = 0.011), both variables considered as continuous variables, were predictive for local control. Interestingly, all local recurrences occurred in colorectal metastases (Table 2). The 2-year actuarial LC rate for PTV prescription BED_10_ > 86.1 Gy_10_ was 96.6 % and for ≤ 86.1 Gy_10_ 68.1 % (Fig. 3), the difference being significant (*p* = 0.005). In multivariate analysis PTV prescription BED_10_ seemed to be the dominating factor for LC, though not reaching significance (*p* = 0.069). However, the results of the multivariate analysis should be interpreted with caution due to the limited number of events (*n* = 10) and all events occurring in colorectal cancer patients harboring the risk of over fitting.

**Table 2:** Univariate and multivariate analysis for local control, progression free interval and overall survival according to patient and tumor characteristics

	Univariate Analysis		Multivariate Analysis	
	HR (CI)	*p*-value	HR (CI)	*p*-value
Local control				
Prior Therapy	1.38 (0.36–5.33)	0.642		
Histology ^a^	<0.001 (0-Inf)	< 0.001	< 0.001 (0-Inf)	0.998
GTV Volume ^b^	0.96 (0.90–1.02)	0.163		
PTV Prescription BED ^b^	0.95 (0.92–0.98)	0.002	0.961 (0.921–1.003)	0.069
GTV Mean BED ^b^	0.975 (0.954–0.996)	0.011	0.995 (0.973–1.017)	0.664
Progression Free Interval				
Karnofsky Index ^d^	0.51 (0.27–0.98)	0.041	0.81 (0.38–1.73)	0.588
Gender	0.79 (0.43–1.46)	0.459	n/a	n/a
Age	0.79 (0.43–1.44)	0.435	n/a	n/a
Histology ^a^	1.20 (0.65–2.21)	0.564	n/a	n/a
Prior Therapy	2.10 (1.04–4.23)	0.034	1.57 (0.73–3.37)	0.244
Extra-hepatic Disease Status ^c^	3.05 (1.59–5.84)	0.000	2.33 (1.07–5.07)	0.033
Largest GTV Volume ^b^	1.002 (0.998–1.006)	0.283	n/a	n/a
Cumulative GTV Volume ^b^	1.003 (0.999–1.007)	0.110	n/a	n/a
Number of Treated Metastases	1.77 (0.92–3.41)	0.082	1.19 (0.84–1.76)	0.386
Overall Survival				
Karnofsky Index ^d^	0.78 (0.37–1.62)	0.496	n/a	n/a
Gender	0.64 (0.31–1.29)	0.204	n/a	n/a
Age	0.78 (0.38–1.58)	0.483	n/a	n/a
Histology ^a^	0.74 (0.37–1.48)	0.396	n/a	n/a
Prior Therapy	1.86 (0.83–4.17)	0.124	n/a	n/a
Extra-hepatic Disease Status ^c^	2.64 (1.30–5.38)	0.005	2.59 (1.26–5.32)	0.009
Largest GTV Volume ^b^	1.003 (0.998–1.008)	0.227	n/a	n/a
Cumulative GTV Volume ^b^	1.004 (0.999–1.009)	0.067	1.004 (0.999–1.009)	0.096
Number of Treated Metastases	1.29 (0.62–2.67)	0.495	n/a	n/a

*GTV* Gross Tumor Volume, *PTV* Planning Target Volume, *BED* Biological Effective Dose, *HR* hazard ratio, *CI* 95 % confidence interval

^a^ Colorectal Cancer vs. Other Primary Cancer

^b^ Evaluated as Continuous Variables

^c^ No Evidence of Diseases vs. Stable or Progressive Extra-hepatic Diseases

^d^ ≤ 80 % vs. 90–100 %

### Progression-free interval

Progression-free interval (PFI) analysis was based on treated patients (*n* = 52). Twenty-eight patients (53.8 %) developed new intrahepatic metastases. Eight of these patients were re-treated locally (six with SBRT). Twenty-four patients (46.2 %) developed extrahepatic metastases, three of whom were treated locally (all with SBRT). The median progression-free interval was 9 months (range, 1–36 months) and the 1- and 2-year actuarial progression-free interval rates were 35.1 and 17.7 %, respectively (Fig. 2). In univariate analysis patient age at time of SBRT, gender, tumor histology, number of treated metastases per patient, and GTV volume (largest metastases or cumulative volume) were not predictive for PFI (Table 2). On the other hand, status of extrahepatic disease at time of SBRT (HR 3.05, CI 1.59–5.84, *p* < 0.001), Karnofsky Index (HR 0.51, CI 0.27–0.98, *p* = 0.041), and prior liver therapy (HR 2.10, CI 1.04–4.23, *p* = 0.034) had significant effects on the PFI in our analysis. Multivariate analysis showed only the status of extra-hepatic disease at time of SBRT to be a significant factor for PFI (HR 2.33, CI 1.07–5.07, *p* = 0.033).

### Overall survival

Overall survival (OS) analysis was based on treated patients (*n* = 52). Median overall survival at time of analysis was 23 months and cause of death was further tumor progression in 91.2 %. Actuarial 1- and 2-year overall survival rates were 70.2 and 45.0 %, respectively (Fig. 2). In univariate analysis patient age at time of SBRT, gender, Karnofsky-Index, number of treated metastases, prior liver therapy and GTV volume (largest metastases or cumulative volume) were not predictive for OS (Table 2). The 2-year actuarial OS was better for patients with colorectal cancer as the primary tumor site (58.7 %) compared to other primary tumor sites (35.7 %), although the difference was not statistically significant (*p* = 0.364). The only statistically significant variable prognostic for OS on univariate (HR 2.64, CI 1.30–5.38, *p* = 0.005) and multivariate (HR 2.59, CI 1.26–5.32, *p* = 0.009) analyses was the status of extrahepatic disease (no evidence of disease vs. stable or progressive extrahepatic disease) at time of SBRT (Fig. 2).

### Toxicity

One patient developed an infected encapsulated hematoma in the liver and a right-sided pleural effusion after fiducial implantation. Overall, radiation treatment itself was well tolerated. Grade 1 acute nausea or fatigue were observed in 24.1 % of the patients and nausea was generally handled with antiemetic. Only one patient (1.9 %) had a grade ≥ 2 side effect and required a stent implantation for hepatic vein occlusion after SBRT of a tumor attached to that vein.

## Discussion

Currently, there is no consensus on how to prescribe the dose and optimize the dose distribution within the gross tumor volume (GTV) or even the planning target volume (PTV) for SBRT treatments. Current Radiation Therapy Oncology Group (RTOG) clinical trial protocols even allow for a considerable inhomogeneity of dose within the PTV, as long as the PTV prescription dose is maintained. Specifically, the PTV-encompassing isodose line in some protocols may range from 60–90 %, giving rise to a possible dose maximum difference of almost 50 % within the same patient cohort. With the focus on the PTV-encompassing isodose line in such approaches, considerable differences in dose distribution in different GTVs may even occur within the same patient! This becomes especially problematic with intensity modulation or in the case of CyberKnife with small non-isocentric non-coplanar beam arrangements. Therefore, it is currently impossible to compare reported dose prescription in the literature, as inhomogeneity is generally not explicitly reported.

The treatment planning approach in our institutions has been to prescribe the dose to the PTV as well, but additionally to optimize the dose distribution in order to achieve a concentric dose build-up and a high-dose plateau to maximize the dose within the GTV. The present analysis was designed to evaluate the efficacy and safety of our GTV-mean-dose optimized treatment planning method for liver metastases where we allowed the reduction of the PTV encompassing prescription dose to adhere to safe normal tissue constraints using the robotic CyberKnife system. Due to the small CyberKnife beams, a ring-like dose distribution with cold spots in the middle can arise when the center of the PTV is not explicitly optimized [24, 32]. This is especially true for lesions with larger GTV-PTV margins such as liver metastases (8 mm in our case) and when inhomogeneous dose prescriptions are used as is typically the case in SBRT treatments.

Our local control rates are well in line with previous publications [15–21, 24, 32–36]. In agreement with other studies [24, 33–35], we found that the PTV prescription BED_10_ was a significant factor for local control. Additionally, we also found a significant influence of mean GTV BED_10_ on local control (Table 2), which has not been demonstrated before. The 2-year actuarial local control was 100 % for PTV > 100.0 Gy_10_ vs. 71.5 % for PTV ≤ 100.0 Gy_10_ (*p* = 0.009) and 96.6 % for PTV > 86.1 Gy_10_ vs. 68.1 % for PTV ≤ 86.1 Gy_10_ (*p* = 0.005). Lanciano et al. [33] and Dewas et al. [34] have reported both substantially lower 2-year local control rates even for doses above 100.0 Gy_10_ and Chang et al. [35] reported the need for 116.5–142.1 Gy_10_ PTV dose to achieve > 90 % local control in similar patient cohorts to ours. On the other hand, our results match those from Stintzing et al. [36] in which they used a PTV prescription dose of 81.6–93.6 Gy_10_, yet for smaller lesions as compared to ours. Unfortunately, most published studies for CyberKnife liver SBRT [24, 32–36] only report the PTV prescription dose and no details on the dose distribution within the PTV or GTV, therefore, a direct comparison to our data is difficult. Although the existence of strong dose-response relationship has been consistently reported, a clear conclusion on the minimally required PTV prescription dose to achieve a certain level of local control remains unknown. Distinct description and reporting of the dose distribution and benchmark trials [29, 37] are necessary for multi-institutional multi-technology comparison or pooled evaluation of SBRT for liver metastases.

Also in agreement with other publications [24, 36], we found significantly better local control for liver metastases originating from non-colorectal cancer (CRC), as all local recurrences in our patient cohort occurred only in CRC patients. In total, three patients with overall ten liver metastases developed local recurrences within the first year after SBRT. However, larger patient numbers are needed to reliably model tumor control probability with regard to histology. Still, as far as CRC liver metastases are concerned, it remains speculative whether the observed inferior local tumor control is due to a differing radio sensibility or due to the observed difference in PTV prescription BED_10_. While the mean GTV BED_10_ was comparable between CRC on non-CRC metastases, the median PTV prescription BED_10_ differed significantly between the two groups (75 Gy_10_ vs. 90 Gy_10_, respectively). Nevertheless, local control for CRC metastases would only reach 80 % even for PTV prescription BED_10_ > 90 Gy_10_, thus a possible effect of different radio sensitivity cannot be excluded. Interestingly, a recent pooled analysis of SBRT for lung metastases did not find a dose-dependent difference in local tumor control for CRC and non-CRC histology [38]. On the other hand, Ahmed et al. have reported on different radiation sensibility of CRC metastases depending on anatomical location, e.g. lung versus liver [39]. Taking other published results and ours into account, dose escalation beyond a prescription PTV BED_10_ greater than 112.5 Gy_10_ (corresponding to 3 × 15 Gy) and a mean GTV BED_10_ greater than 151.2 Gy_10_ (corresponding to 3 × 18 Gy) for non-CRC metastases does not seem necessary.

GTV volume (either based on largest metastases or on cumulative volume) was not a significant factor for local control in our patient cohort. The literature however is controversial regarding tumor size being a factor for local control after SBRT as some studies found this to be significant [34, 40] while others did not [17, 19, 36]. A speculation about the treatment planning technique as being one reason for the significant or non-significant influence of GTV volume on local control is beyond the scope of this paper. Still, we hypothesize under the assumption of the existence of a GTV dose-volume effect that the high mean GTV BED_10_ may have annihilated this effect. Furthermore, we hypothesize based on our results that an optimization with reduced PTV prescription dose (if necessary for adherence to normal tissue constraints) will not result in significant inferior local control if a minimum PTV encompassing BED_10_ of greater 86.1 Gy_10_ and a high GTV mean BED_10_ of greater 151.2 Gy_10_ is maintained.

Overall survival of 45.0 % at 2 years was comparable to published literature [16–20, 32–36] and comparable to other local therapy modalities [11–14, 41, 42]. In our cohort, patients with CRC had better overall survival at 2 years after SBRT compared to those with other primary tumors (58.7 vs. 35.7 %), although the result was not statistically significant. Andratschke et al. [17] and Rusthoven et al. [43] have reported a significant influence of CRC histology on the overall survival while Dewas et al. [23] and Rule et al. [44] did not find that overall survival was dependent on primary tumor site. On the other hand, in our analysis the status of extra-hepatic disease at time of SBRT was the most influencing factor on overall survival in agreement with other studies [17, 35]. Patients with no evidence of extrahepatic disease had significantly better overall survival and progression-free intervals.

In our study, acute toxicities were minimal and consisted of grade 1 fatigue, nausea and vomiting in 24.1 % of the patients. Only 1 patient (1.9 %) had a late toxicity of grade ≥ 2 requiring a stent implantation after hepatic vein occlusion. Our toxicity data compare favorably well with published reports as they range at the lower spectrum of reported side effects [15–20, 32–36].

Limitations to our findings are inherent to the retrospective nature of our study, even though we treated them according to study-like institutional guidelines. Although, a larger number of metastases were evaluated, the combined analysis of different tumor histologies with various prior treatments makes thorough determination of predictive factors difficult. Furthermore, with the low number of events, especially for local control (*n* = 10), serious conclusions from the multivariate analysis cannot be drawn. Larger patient cohorts, either collected as a multi-institutional registry or ideally enrolled in a prospective study with coherent patient criteria, longer follow-up periods and detailed patient and dosimetry information are needed in order to validate our assumptions and to define the patient groups significantly to benefit from SBRT.

## Conclusions

Overall, robotic real-time-motion-compensated SBRT can be an effective and safe treatment with minimal toxicities and high local tumor control rates. As long as GTV mean BED_10_ greater 151.2 Gy_10_ is maintained, a significantly lower PTV prescription BED_10_ compared to common published literature can be sufficient for very high local control rates. Nevertheless, it seems that even with GTV mean dose optimization, a reasonable minimum PTV prescription BED_10_ of greater than 86.1 Gy_10_ is required. Further analyses of optimal treatment planning, dose schedules and delivery technique for liver SBRT is required. In the end, extra-hepatic disease status remains the major independent factor for overall survival and may guide the decision for intensive local treatment in this patient cohort.

## Abbreviations

BED: biological effective dose
CI: confidence interval
CRC: colorectal cancer
CT: computer tomography
CTV: clinical target volume
GTV: gross target volume
HR: hazard ratio
LC: local control
MRI: magnetic resonance imaging
OS: overall survival
PFI: progression-free interval
PTV: planning target volume
SBRT: stereotactic body radiation therapy
